# Enhancing nnUNetv2 Training with Autoencoder Architecture for Improved Medical Image Segmentation

**DOI:** 10.1007/978-3-031-83274-1_17

**Published:** 2025-03-03

**Authors:** Yichen An, Zhimin Wang, Eric Ma, Hao Jiang, Weiguo Lu

**Affiliations:** 1 NeuralRad LLC, Madison, WI, USA; 2 Department of Radiation Oncology, UT Southwestern Medical Center, Dallas, TX, USA

**Keywords:** MRI-guided radiotherapy, nnUNetv2, Autoencoder, Deep learning, Head and neck cancer, Tumor segmentation, Dice similarity coefficient, Medical image segmentation

## Abstract

Auto-segmentation of gross tumor volumes (GTVs) in head and neck cancer (HNC) using MRI-guided radiotherapy (RT) images presents a significant challenge that can greatly enhance clinical workflows in radiation oncology. In this study, we developed a novel deep learning model based on the nnUNetv2 framework, augmented with an autoencoder architecture. Our model introduces the original training images as an additional input channel and incorporates an MSE loss function to improve segmentation accuracy. The model was trained on a dataset of 150 HNC patients, with a private evaluation of 50 test patients as part of the HNTS-MRG 2024 challenge. The aggregated Dice similarity coefficient (DSCagg) for metastatic lymph nodes (GTVn) reached 0.8516, while the primary tumor (GTVp) scored 0.7318, with an average DSCagg of 0.7917 across both structures. By introducing an autoencoder output channel and combining dice loss with mean squared error (MSE) loss, the enhanced nnUNet architecture effectively learned additional image features to enhance segmentation accuracy. These findings suggest that deep learning models like our modified nnUNetv2 framework can significantly improve auto-segmentation accuracy in MRI-guided RT for HNC, contributing to more precise and efficient clinical workflows.

## 1 Introduction

Radiation therapy (RT) is a cornerstone in the treatment of head and neck cancer (HNC), and recent advances in MRI-guided RT have significantly improved treatment precision. The superior soft tissue contrast of MRI allows for better tumor delineation compared to conventional CT imaging, leading to enhanced treatment planning [1]. However, manual tumor segmentation is both time-consuming and error-prone due to the complex anatomy of HNC tumors [2]. This challenge has led to the increased use of artificial intelligence (AI) in automatic tumor segmentation. Deep learning, specifically through the nnU-Net framework [3], has shown tremendous potential in addressing the segmentation challenges in RT planning. The HNTS-MRG 2024 challenge, focused on head and neck tumor segmentation, encourages participants to leverage MRI data to develop robust segmentation algorithms for pre- and mid-radiotherapy images. This challenge offers a unique opportunity to evaluate how incorporating multi-time point data can improve segmentation outcomes. In this work, we present a novel approach utilizing a nnUNet-based model with an autoencoder architecture to address these segmentation challenges. By introducing additional input channels, such as training images, and incorporating mean squared error (MSE) loss, we aim to enhance segmentation accuracy. Our model was trained over multiple folds and demonstrated improved performance over the baseline nnUNet model, achieving promising results after 1000 epochs and seven stages of training. This paper details our approach and evaluates its effectiveness in the HNTS-MRG 2024 challenge.

## 2 Related Works

The nnU-Netv2 framework [3] has become a leading solution for medical image segmentation due to its robust, self-adapting architecture. It automatically configures its hyperparameters based on the dataset, making it highly versatile across different segmentation tasks. nnU-Net’s core design includes both 2D and 3D U-Net models, offering flexibility for a range of medical imaging modalities. A key feature is its dynamic patch size adjustment and depth scaling, allowing the architecture to adapt to specific tasks without manual intervention. Previously, our group developed a nnUNet-based platform for automatic delineation of Head & Neck GTV, which ranked No. 3 in the HECKTOR 2022 challenge [4]. Recent developments, such as the **Residual Encoder Presets** [5] introduced in nnUNetv2, have shown improvements in segmentation performance by incorporating deeper encoder stages (up to seven) to extract more detailed feature representations. These presets integrate residual connections, which enhance the model’s ability to retain learned information across layers, addressing the vanishing gradient problem. By using **7-stages** in our task, we leverage these architectural improvements to achieve more accurate segmentation results, particularly in multi-channel settings, such as incorporating auto-encoder predictions along with traditional segmentation targets.

This evolution of the nnU-Net framework demonstrates its continuous adaptability and high performance, making it a fitting choice for the HNTS-MRG 2024 challenge. In this challenge, we apply these advances to segment head and neck tumors in MRI images, targeting both pre- and mid-radiotherapy scans to optimize radiation therapy workflows.

## 3 Methods

The model training was conducted on an Intel i9–14900 CPU and an Nvidia GeForce RTX 4090 GPU with 24 GB memory. The training process spanned 1000 epochs, with each epoch consisting of 250 iterations and 50 validation iterations. Stochastic Gradient Descent (SGD) was employed as the optimizer, initialized with a learning rate of 1e-2, momentum set to 0.99, and a weight decay of 3e-5. Deep supervision was applied during training, along with an oversampling strategy featuring a foreground sampling percentage of 33%. Additionally, five-fold cross-validation was utilized to ensure robustness and generalizability, optimizing the model’s ability to handle complex computations and large datasets effectively across all epochs.

**Fig. 1.**
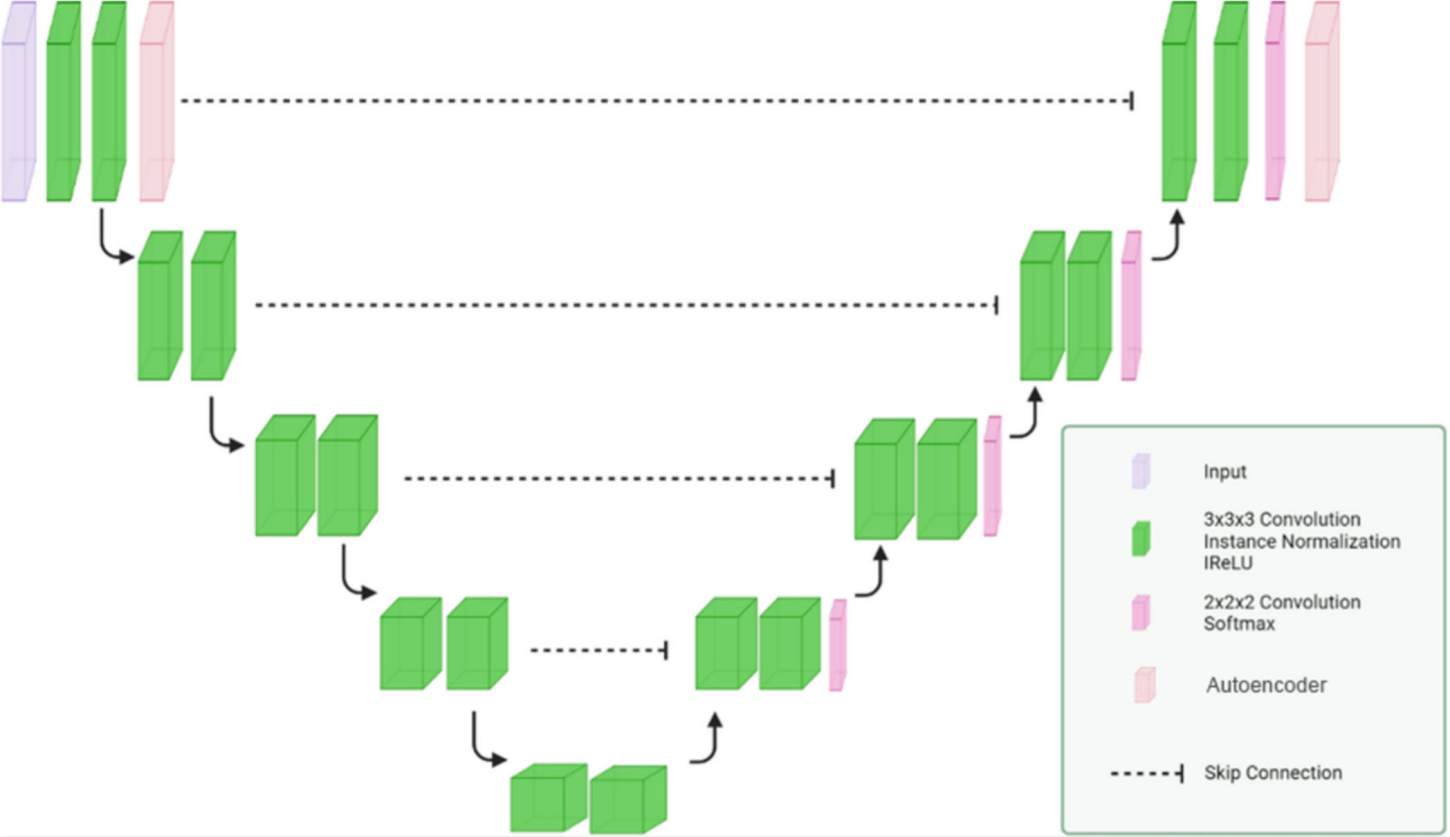
Autoencoder layer for our nnUNetv2

The image processing pipeline is built upon nnUNetv2’s framework with both standard and custom modifications. The dataset was preprocessed using fingerprint information, including voxel spacing, intensity normalization, and size standardization, ensuring consistency across inputs (in Fig. 1). All images were cropped to their non-zero regions during preprocessing, resampled to isotropic spacing, and normalized to a fixed intensity range. Additionally, a comprehensive voxel connectivity analysis was conducted to identify and remove isolated noise artifacts erroneously labeled as tumor regions. These refinements enhanced the dataset’s quality for more accurate downstream analysis.

A critical modification introduced was the inclusion of original input data as an additional input channel for training. Specifically, the original training images parsed from nnUNetv2 were stored and passed into the training process without concatenation. This stored image data was processed separately during autoencoder training to improve segmentation accuracy. Instead of being merged with other channels, the original image was preserved as its own channel and treated as an auxiliary target in the autoencoder structure. For instance, a tensor originally shaped as [3, 67, 512, 512] (representing three segmentation targets) was transformed into [4, 67, 512, 512], where the additional channel corresponds to the original image data. This transformation enables the model to jointly process segmentation labels and input image data for enhanced feature learning. During the data loading phase, the pipeline was extended to allow storage and utilization of the parsed image data as an additional custom input channel. Moreover, the target format was modified to accept 32-bit floating-point (float32) data rather than the default 16-bit integer (int16) format, ensuring compatibility with our extended training pipeline. This adjustment was critical for integrating the mean squared error (MSE) loss function, which requires continuous floating-point data for accurate gradient computation. The enhanced pipeline enabled the model to optimize for both segmentation and image reconstruction in a unified framework.

Specifically, we assert that the predicted output and target shapes match, applying the MSE loss as:

(1) $$\text{MSE}=\frac{1}{N}\sum_{i=1}^{N}{\left(x_{i}-y_{i}\right)}^{2}$$

(2) $$\text{Dice Loss}=1-\frac{2\times \sum (P\times G)}{\sum P+\sum G}$$

To enhance the model’s performance, we introduced a compound loss function, combining Dice loss for segmentation and MSE for autoencoder reconstruction (DC_and_MSE_loss). This function separates the final image layer for MSE loss calculation, while the remaining layers compute the segmentation loss via Dice. This dual-objective approach ensures both segmentation accuracy and image reconstruction quality in training.


(3) L=1.2×MSE+1.0×Dice

**Fig. 2.**
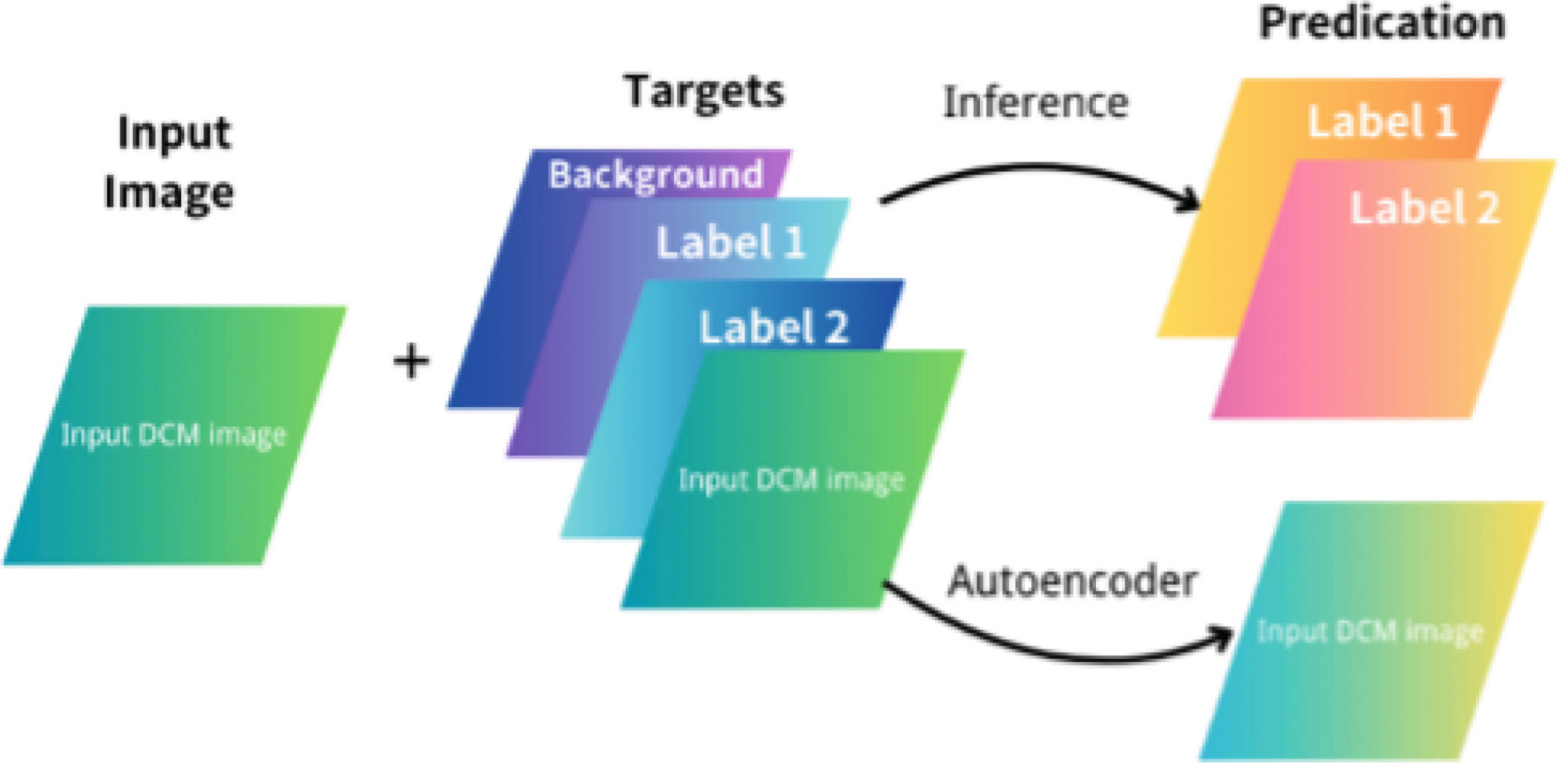
Input/Output layers

During the inference phase, we configured the autoencoder with an additional output channel dedicated to predicting the original input images. This added output channel allows the model to generate both segmentation results and auto-encoder predictions simultaneously. As shown in Fig. 2, the structure of the network ensures that the auto-encoder prediction is handled as a separate task, which enables better alignment between the predicted auto-encoder image and the segmented regions, supporting improved overall performance in tasks requiring auto-encoder images and segmentation outputs.

## 4 Results

**Fig. 3.**
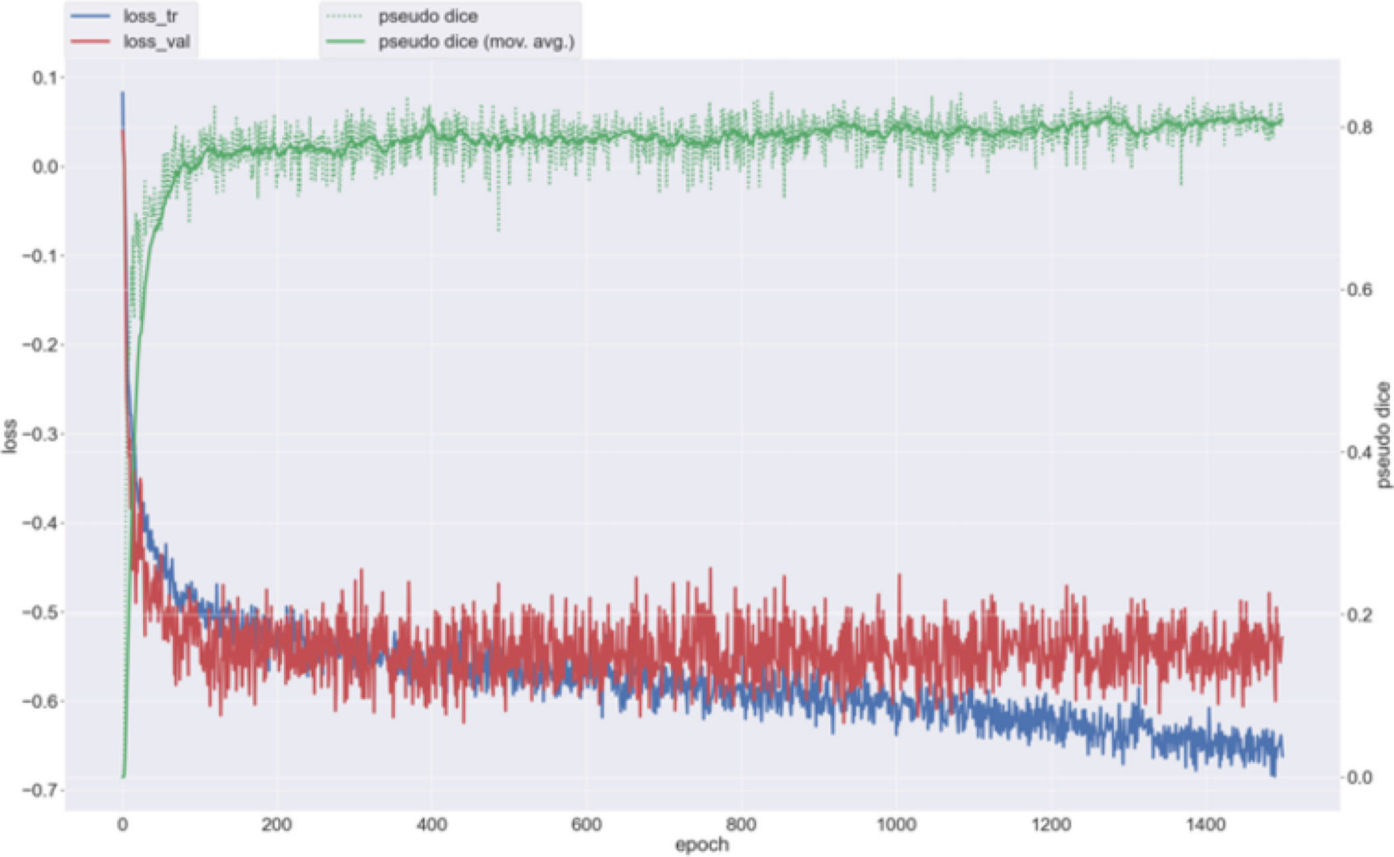
Learning curve

The learning curve indicates stable model performance over 1000 epochs, as shown in Fig. 3. Both training and validation losses decrease rapidly during the initial training stages and plateau after approximately 600 epochs. This pattern suggests minimal overfitting and highlights the model’s ability to generalize well across the dataset. Simultaneously, the pseudo-Dice score shows a steady improvement, stabilizing after around 600 epochs, further demonstrating the robustness of the model’s learning process.

**Fig. 4.**
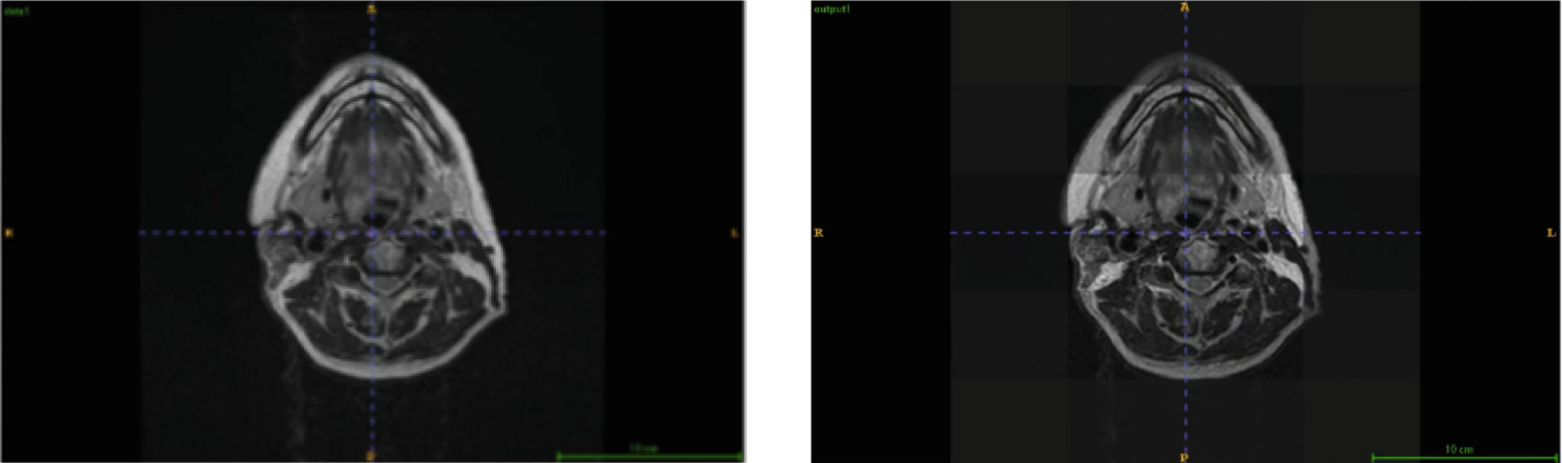
Original Input Image vs Auto-encoder Output

**Fig. 5.**
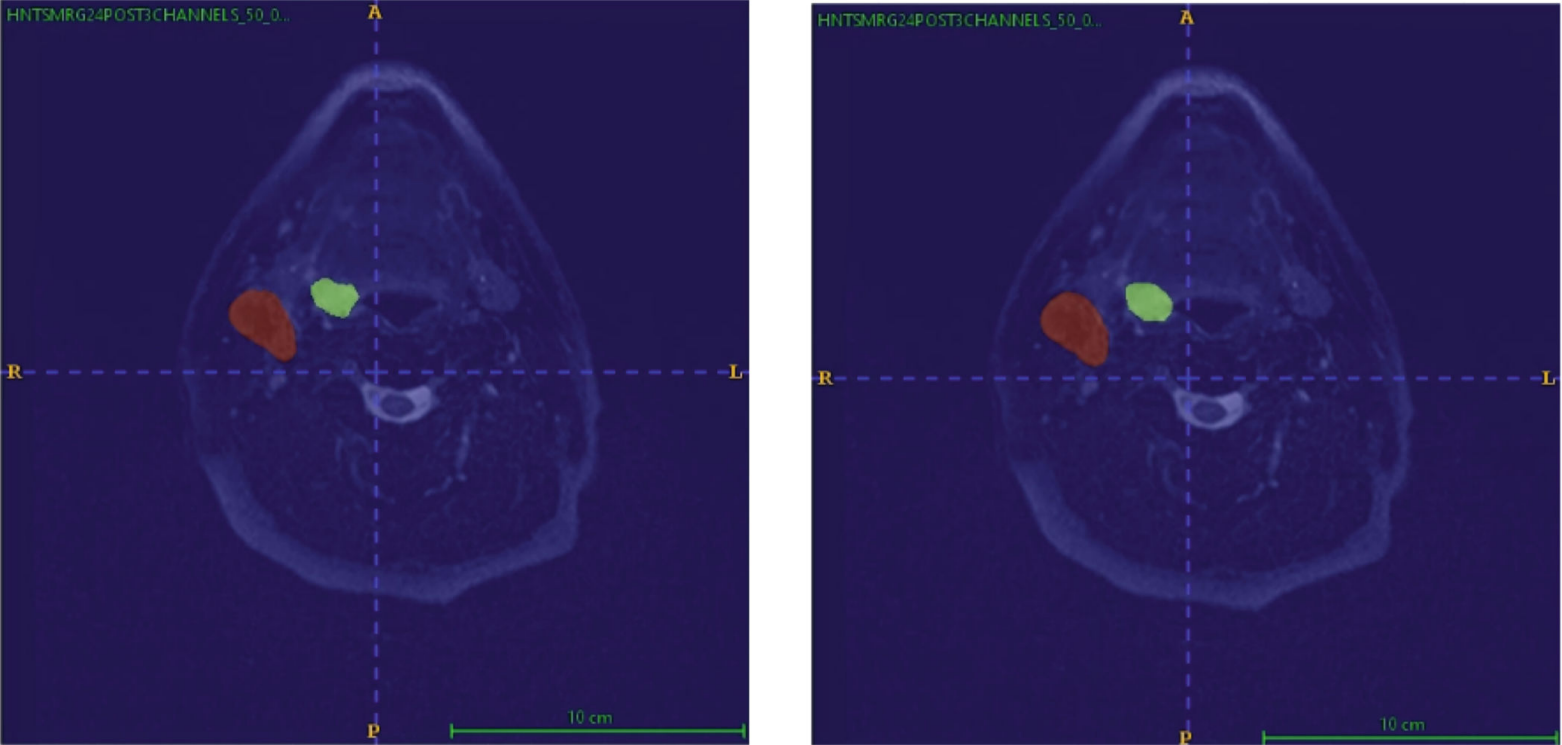
Ground Truth Label vs Prediction with nnUNetv2 7-stages

In our experiment, the input image was reconstructed alongside the segmentation task. As depicted in Fig. 4, the reconstructed image closely resembles the original input, demonstrating the autoencoder’s capacity to capture image features effectively. This indicates that the autoencoder positively contributes to feature extraction, ultimately enhancing the learning process. Additionally, Fig. 5 highlights the modified nnUNetv2’s strong predictive capabilities, with the model accurately identifying the target labels.

The final results from the private evaluation phase of the HNTS-MRG 2024 challenge, involving 50 test patients, confirm the model’s promising performance. Using the aggregated Dice Similarity Coefficient (DSCagg) metric, the metastatic lymph nodes (GTVn) achieved a DSC of 0.8516, while the primary tumor (GTVp) scored 0.7318. The mean DSCagg across both structures was 0.7917. These findings underscore the model’s robust performance in segmenting lymph nodes while revealing areas for improvement in primary tumor segmentation. Future iterations of the model will focus on addressing this discrepancy to achieve more balanced performance across all target structures.

## 5 Discussion

The autoencoder’s ability to replicate the input image in a nearly identical manner showcases its potential to improve model performance. By learning the input features through reconstruction, the encoder strengthens the feature extraction process in the segmentation network. This additional representation learning allows the network to generalize better across different datasets, improving segmentation accuracy in complex tasks such as head and neck cancer segmentation. The use of MSE loss in combination with the Dice loss ensures both accurate segmentation and feature retention, benefiting the overall model performance.
